# *Babesia microti* alleviates disease manifestations caused by *Plasmodium berghei* ANKA in murine co-infection model of complicated malaria

**DOI:** 10.3389/fcimb.2023.1226088

**Published:** 2023-07-10

**Authors:** Iqra Zafar, Tomoyo Taniguchi, Hanadi B. Baghdadi, Daisuke Kondoh, Mohamed Abdo Rizk, Eloiza May Galon, Shengwei Ji, Shimaa Abd El-Salam El-Sayed, Thom Do, Hang Li, Moaz M. Amer, Ma Zhuowei, Ma Yihong, Jinlin Zhou, Noboru Inoue, Xuenan Xuan

**Affiliations:** ^1^ National Research Center for Protozoan Diseases, Obihiro University of Agriculture and Veterinary Medicine, Obihiro, Japan; ^2^ Livestock and Dairy Development Department, Veterinary Research Institute, Lahore, Punjab, Pakistan; ^3^ Department of Immunology and Parasitology, Graduate School of Medicine University of the Ryukyus, Nishihara Cho, Japan; ^4^ Biology Department, College of Science, Imam Abdulrahman Bin Faisal University, Dammam, Saudi Arabia; ^5^ Basic and Applied Scientific Research Center (BASRC), Imam Abdulrahman Bin Faisal University, Dammam, Saudi Arabia; ^6^ Department of Veterinary Medicine, Obihiro University of Agriculture and Veterinary Medicine, Obihiro, Japan; ^7^ Department of Internal Medicine and Infectious Diseases, Faculty of Veterinary Medicine, Mansoura University, Mansoura, Egypt; ^8^ College of Veterinary Medicine and Biomedical Sciences, Cavite State University, Indang, Cavite, Philippines; ^9^ Department of Biochemistry and Chemistry of Nutrition, Faculty of Veterinary Medicine, Mansoura University, Mansoura, Egypt; ^10^ Shanghai Veterinary Research Institute, Chinese Academy of Agricultural Sciences, Shanghai, China

**Keywords:** *Babesia microti* Peabody mjr, *Plasmodium berghei* ANKA, co-infection, cross immunity, malaria, babesiosis, innate immunity, acute stage immunity

## Abstract

Malaria remains one of the most significant health issues worldwide, accounting for 2.6% of the total global disease burden, and efforts to eliminate this threat continue. The key focus is to develop an efficient and long-term immunity to this disease via vaccination or therapeutic approach, and innovative strategies would enable us to achieve this target. Previously, using a mouse co-infection disease model, cross-protection was illustrated between *Babesia microti* and *Plasmodium chabaudi*. Hence, this study was planned to elucidate the impact of acute *B. microti* Peabody mjr and *Plasmodium berghei* ANKA co-infection on the consequence of complicated malaria in the C57BL/6J mouse model of malaria. Furthermore, immune response and pathological features were analyzed, and the course of the disease was compared among experimental groups. Our study established that acute *B. microti* infection activated immunity which was otherwise suppressed by *P. berghei*. The immunosuppressive tissue microenvironment was counteracted as evidenced by the enhanced immune cell population in co-infected mice, in contrast to *P. berghei*-infected control mice. Parasite sequestration in the brain, liver, lung, and spleen of co-infected mice was significantly decreased and tissue injury was ameliorated. Meanwhile, the serum levels of IFN-γ, TNF-α, and IL-12p70 were reduced while the secretion of IL-10 was promoted in co-infected mice. Eventually, co-infected mice showed an extended rate of survival. Hereby, the principal cytokines associated with the severity of malaria by *P. berghei* infection were TNF-α, IFN-γ, and IL-12p70. Moreover, it was evident from our flow cytometry results that innate immunity is crucial and macrophages are at the frontline of immunity against *P. berghei* infection. Our study recommended further investigations to shed light on the effects of babesiosis in suppressing malaria with the goal of developing *Babesia*-based therapy against malaria.

## Introduction

Despite countless efforts towards the elimination of malaria, it is endemic in many countries and accounts for more than 200 million cases of infection every year; 619,000 malaria deaths were estimated in 2022 (World Malaria Report, 2022). Due to climate change, a spike in the geographical dissemination of malaria in tropical regions is predicted (Cella et al., 2019). The agent responsible for malaria is the intracellular parasite *Plasmodium*; it enters the host through the bite of an infected vector, *Anopheles* mosquitoes (Sanches-Vaz et al., 2019). Five *Plasmodium* species can infect humans; among these species, *Plasmodium falciparum* is exceptionally lethal as it can result in clinical symptoms that vary from asymptomatic to severe manifestations of the disease (Hunt et al., 2006; Mishra & Newton, 2009). The notion of “severe malaria” includes a diverse range of syndromes in humans, such as acute respiratory distress (pulmonary edema), hyperparasitemia, severe anemia, liver dysfunction, acute renal failure, hypoglycemia, multiorgan failure, and eventually, cerebral malaria (CM), with a mortality of 15–20% (Dondorp et al., 2000; De Oca et al., 2013). Pulmonary edema, acute renal failure, and jaundice are frequent in adults, whereas anemia, hypoglycemia, and convulsions prevail in children (Dondorp et al., 2000). Hematological alterations implicated with malaria comprise anemia, thrombocytopenia, and disseminated intravascular coagulopathy. Involvement of the liver is frequent in patients with complicated malaria; clinical symptoms include jaundice, liver enlargement, and high levels of liver enzymes such as aspartate and alanine transaminases (Al-Salahy et al., 2016). Cerebral malaria is catastrophic from these disease outcomes (Ghazanfari et al., 2018), and is mostly found in children <5 years of age with morbidity and mortality of 15 to 20%. Even if children survive they are affected with lifelong neurological sequelae (Pais et al., 2022). At present, there is no remedy for CM, hence the study of host and parasite interactions to understand the pathogenesis is of utmost importance for designing new therapeutic interventions (Mineo et al., 2013; Briquet et al., 2015).

Infection of C57BL/6J mice with *Plasmodium berghei* ANKA has been considered as a model for experimental cerebral malaria (ECM), as it reproduces most of the pathologies identified in complicated malaria (De Oca et al., 2013). Infiltration of infected red blood cells (iRBCs) in the brain and lung microvasculature is the characteristic feature of human cerebral malaria (HCM). Although the interaction of iRBC with the endothelium is linked to the intensity of malaria, the infiltration of iRBC by *P. berghei* ANKA has not been completely characterized (El-Assaad et al., 2013). Several other pathophysiological features of severe malaria are not yet understood (Dondorp et al., 2000). The pursuit to eradicate malaria needs further understanding of the pathogenesis of this disease (Mawson, 2013; Junaid et al., 2017). An effective therapeutic strategy against this ailment has not been successfully established. Therefore, it is essential to comprehend the mechanism of protection against *Plasmodium* infection (Yoneto et al., 1999). Intriguingly, protection against malaria can also be obtained from previous parasite infections. By using co-infection mouse models, cases of cross-protection were illustrated between *Babesia* and *Plasmodium* (Miyagami et al., 1987; Efstratiou et al., 2020). Babesiosis is an understudied, tick-transmitted disease of zoonotic importance. The agent responsible for it is the intraerythrocytic parasite *Babesia*, predominantly *Babesia microti*, which culminates in the development of malaria-like disease but that can be asymptomatic (Homer et al., 2000; Elton et al., 2019; Zafar et al., 2022). The host can establish immunity to *Babesia* spp. after infection. Cell-mediated and humoral immunity both participate to protect against babesiosis (Homer et al., 2000; Elton et al., 2019). Cases of mixed infection with *B. microti* and other pathogens are rising, wherein *Babesia* parasites themselves act as immunomodulators (Lobo et al., 2013; Swanson et al., 2023).

A phenomenon where a host establishes immunity against a pathogen after being exposed to a non-identical pathogen is called “heterologous immunity” (Welsh et al., 2010). In developing countries, co-infections are not an exception, rather, the actual clinical picture includes a wide array of pathogens that impact one another in addition to the host (Lundqvist et al., 2010). There is a paucity of information about co-infections and their consequences on health (McArdle et al., 2018). Co-infections with some pathogens may impede disease diagnosis. A deeper understanding of the impact of co-infections on host immune response and disease outcome will augment diagnosis and the development of novel clinical interventions (Mabbott, 2018). In 1983, the first case of co-infection was documented in humans with both *Plasmodium* and *Babesia* parasites (Vermeil et al., 1983). Co-infections by *Babesia* spp. and *Plasmodium* spp. have been recorded in areas where malaria is endemic (Ahn, 2010; Zhou et al., 2013; Na et al., 2014; Gabrielli et al., 2016). In mice, *B. microti* has successfully conferred cross-immunity against *Plasmodium* spp. (Cox, 1972; Zivkovic et al., 1984). Other cases of cross-protection have been observed between *B. microti* and *P. vinckei* (Cox, 1978), *P. berghei* ANKA and *P. berghei* NK65 XAT (Miyagami et al., 1987), and *P. berghei* XAT (Pb XAT) and *P. berghei* NK65 (Pb NK65) (Niikura et al., 2008). *B. microti* was also able to protect from *P. cynomolgi* (Van Duivenvoorde et al., 2010) and *P. chabaudi* (Efstratiou et al., 2020) infections. The sequestration of iRBCs in various organs facilitated by cytoadherence has not yet been recorded in *Babesia*. Antibodies seem to have a minor part in the resolution of these iRBCs, whereas macrophages not only help in the resolution of both infections but also confer cross-protection against these parasites (Djokic et al., 2021). Nevertheless, there is a scarcity of knowledge available on the immunology of co-infections, and the malaria-inhibiting impact of babesiosis is not fully understood (Efstratiou et al., 2020). The development of a malaria vaccine is among the foremost goals in biomedical research. RTS,S, is a subunit vaccine derived from *P. falciparum* circumsporozoite protein (CSP) (Nunes-Cabaço et al., 2022). However, RTS,S does not provide protection against the remaining malaria parasites, specifically *P. vivax*, *P. ovale*, *P. malariae*, and *P. knowlesi* (Venkatesan, 2022). To date, protein subunit- and/or DNA-based vaccines have shown restricted efficacy and disappointing results (Douradinha et al., 2008). An alternative form of vaccine is the whole-organism vaccine. Immunization using complete irradiated sporozoites stands out as one of the most efficacious vaccinations against the same parasite strains, providing 100% protection. Nonetheless, irradiated sporozoites exhibit reduced effectiveness when confronted with heterologous parasite strains. Unlike liver-stage vaccines, the utilization of a blood-stage genetically attenuated malaria parasite (GAP) remains unavailable for human application. Nevertheless, there are advantages to utilizing whole-pathogen vaccines. For instance, whole organism vaccines encompass the complete array of parasite proteins, whereas subunit vaccines generally consist of only one or a few specific target proteins. As a result, if the predominant malaria parasite goes through a mutation in the gene targeted by the subunit vaccine, the vaccine is prone to lose its efficacy. In contrast, a whole-organism vaccine will maintain its stability against single gene mutations (Imai et al., 2020). There is a dire need for a vaccine that could confer cross-protection against multiple plasmodial species including *P. falciparum* and *P. vivax*. Such a vaccine would yield substantial economic, safety, and manufacturing benefits. However, the cross-species immunity in malaria, particularly concerning the protection provided by live attenuated whole-organism-based approaches, has been disregarded in recent decades. Unfortunately, none of the documented cases of human co-infections managed to identify the pathogens beyond the species level. Therefore, the effect of pathogen genetic background on subsequent pathogenicity is not known (Efstratiou et al., 2020). To conclude, it is imperative to thoroughly examine and assess the potential for inducing cross-species immunity by utilizing the whole-organism approach in designing malaria vaccines. In light of this objective, future research is warranted to investigate the immunological mechanisms responsible for eliciting and sustaining cross-protection by employing heterologous parasites (Douradinha et al., 2008).

It is worth noting, however, that co-infection of *B. microti* Peabody mjr (ATCC® PRA-99™), a human isolate, and *P. berghei* ANKA employing C57BL/6J murine model has not been studied so far. In light of the above information, we postulated that primary *B. microti* infection during the acute stage might influence the susceptibility of mice to *P. berghei* ANKA challenge infection. Therefore, in the quest to decipher the host immune mechanism and to strengthen the knowledge of host-parasite interactions in a co-infection model, the current study was performed wherein we highlighted the immune dynamics and various aspects of disease manifestations in a co-infection disease model best suited for complicated malaria.

## Materials and methods

### Experimental animals

To analyze co-infection and experimental cerebral malaria (ECM), 5-week-old female C57BL/6J mice were bought from CLEA, Japan. To avoid any discrepancies that may arise due to the effect of testosterone, female mice were used (Djokic et al., 2019).

### Parasite maintenance and experimental design

Cryostabilates of *B. microti* Peabody mjr (ATCC® PRA-99™) were obtained from the cell bank. *Plasmodium berghei* ANKA was provided by Nagasaki University with support in part by National BioResource Project (NBRP), MEXT, Japan. After thawing the frozen stabilates, mice were intraperitoneally inoculated to passage and were maintained for experiments. The research was performed to investigate the influence of a non-lethal *B. microti* primary infection on a consequent lethal *P. berghei* infection. One group of mice (n=6) was infected by intraperitoneal injection with 10^8^ *B. microti*-infected red blood cells (iRBCs). Then, the same group was challenge infected with 10^3^ P*. berghei*-infected RBCs at day 7 (henceforth mentioned as Bm/PbA7; n=6). Likewise, one more group (n=6) was intraperitoneally inoculated with 10^3^ P*. berghei*-infected RBCs only (henceforth mentioned as PbA) (n=6) and an additional group with *B. microti*-infected RBCs only (henceforth mentioned as Bm) (n=6). A series of experiments was undertaken to probe the effects of co-infection. Preliminary experiments were conducted to optimize the infection dose. Out of the four doses tested (10^2^,10^3^, 10^4^, 10^5^ iRBCs), the minimum infection dose of 10^3^ iRBCs was selected for *P. berghei* infection. Similarly, for *B. microti* infection, two doses were evaluated during the preliminary trial, and out of those, 10^8^ iRBCs was selected as the primary infection dose (Mordue and Wormser, 2019) (**Supplementary Figure 3**). After preliminary experiments, the initial study was executed with four groups, namely *B. microti*-infected group (Bm), co-infected group (Bm/PbA7), a control group (PbA), and a Naïve mouse group (injected with PBS), to investigate the progression of parasitemia, ECM signs, and other parameters. The subsequent trial was conducted including co-infected (Bm/PbA7), control (PbA), and Naïve groups (n=5) wherein the extent of blood-brain barrier (BBB) disruption and the integrity of the vascular endothelial barrier in the lungs were assessed using the Evans blue assay. Another trial was conducted with Bm, PbA, Bm/PbA7, and Naïve mouse groups (n=5) to determine the parasite burden in tissues and the host immune response against infections. At the end, mice were again infected according to the aforementioned experimental design for performing the histopathological analysis.

### Monitoring mice for parasitemia and hematological parameters

Regular monitoring of mice was done, and parameters including parasitemia and body weight were recorded. RBCs and hematocrit (HCT) values were measured by Celltac Alpha MEK-6550K (Nihon Kohden) (Li et al., 2012). To determine the parasitemia percentage, thin blood smears were made after staining with Giemsa; the percent parasitemia was assessed from 10^3^ iRBCs after examining under 100 × oil immersion Eclipse E200 microscope (Nikon, Tokyo, Japan). As we assessed the parasitemia using blood smears, it was challenging to differentiate the levels of parasitemia specifically for *Babesia* and *Plasmodium* parasites in Bm/PbA7 mice. Consequently, we presented the combined parasitemia levels as an overall representation.

### Assessment of experimental cerebral malaria and survival rates

Mice were monitored daily for survival rates, and symptoms were recorded for the development of ECM by the use of the rapid murine coma and behavior scale (RMCBS) score, as explained by (Carroll et al., 2010). When the RMCBS score was ≤5/20, mice were categorized as exhibiting ECM symptoms (Sanches-Vaz et al., 2019).

### Use of flow cytometry to determine the immunophenotype of organs

In a new trial, brain, liver, lungs, kidney, and spleen were harvested aseptically, weighed, and photographed at day 8 post-challenge infection from Bm/PbA7 mice (day 15 post-primary infection) and at day 8 from PbA, Bm, and Naïve mice (n=5) following *P. berghei*, *B. microti* and PBS inoculation respectively. For single cell suspension, PBS was used for spleen and digest solution was used for rest of the organs (Liu et al., 2020). Then, organs were cut and triturated to make single cell suspension. Ground spleen tissues were strained a in 50 mL tube through a 70 µm nylon sterile cell strainer, whereas other organ tissues were incubated in digest solution for 1 h at 37°C. After digestion, the rest of the organ tissues were strained as described earlier. Single-cell suspensions of liver, lungs, kidney, and spleen were washed with PBS by centrifugation at 375 × g for 5 min at 4°C and RBCs were lysed with 1× ACK lysis buffer (Gibco, Massachusetts, USA) for 5 min at 25°C. After lysis was stopped by adding cold PBS, centrifugation was performed at 375 × g for 5 min at 4°C; 2 mL cell staining buffer (CSB, BioLegend, California, USA) was used to resuspend the cell pellet and maintained at 4°C until the next step. Cell suspension of brain tissues was processed differently. The strained brain tissue suspension was washed with PBS and the pellet was resuspended with 8 mL of 40% Percoll (Cytiva, Tokyo, Japan). Then, 5 mL of 80% Percoll was taken into a round bottom tube and 8 mL of 40% Percoll was layered on top of it. After centrifugation at 1,578 × g for 20 min at 25°C with low acceleration (acceleration 1) and no break, the middle interface layer was collected and transferred into a new tube containing 10 mL PBS. Later, centrifugation was carried out at 375 × g for 5 min at 4°C, and 2 mL CSB was used to resuspend the pellet. Two antibody panels were used for each sample and approximately one million cells were reconstituted in CSB (**Supplementary Tables 1.1**-**1.2**). After, Fc block was performed by resuspending the reconstituted cells with 70 µL CSB containing CD16/CD32 monoclonal antibody (Invitrogen, Massachusetts, USA) at 4°C for 25 min. Later, staining of samples was carried out, wherein the cells were labeled with fluorophore-tagged, marker antibodies for each type of immune cell and incubated at 4°C for 30 min in the dark. Then, samples were fixed with 4% paraformaldehyde (PFA) solution for 15 mins and washed two times with CSB and centrifuged at 375 × g for 5 min at 4°C. Finally, 200 µL CSB was used to resuspend the cells. The above mentioned protocol was described by (Rico et al., 2021). Sorting of stained and fixed cells was conducted by employing the CytoFLEX flow cytometer (Beckman Coulter, California, USA) and data were carefully evaluated by CytExpert 2.4 software (Beckman Coulter, California, USA) (**Supplementary Figure 1**). A representation of the cell sorting strategy is shown in **Supplementary Figure 2**, followed according to (Bayne and Vonderheide, 2013) (Zafar et al., 2022).

### Evaluation of organ damage by histopathology

After harvesting the organs from experimental groups (n=1), the fixation of tissues was done in 4% paraformaldehyde, followed by dehydration in graded alcohol and embedding in paraffin. Next, 5 μm thick slices were made. Subsequently, the sections were deparaffinized and stained using hematoxylin & eosin (H & E). Eventually, the sections were mounted on MGK-S with coverslips. Changes in tissue histology were examined by the histopathologist in a blinded manner, by utilizing a Microphot-FX from Nikon, and images were captured using a Digital Sight DS-5M camera also from Nikon.

### Quantification of serum cytokine levels

All groups of mice (Bm, PbA, Bm/PbA7, and Naïve n=5) were exsanguinated by withdrawing the blood from the heart at day 8 post-primary infection or day 8 pci with *P. berghei*. Later, the serum was collected by centrifugation at 1,500 × g for 15 mins at 4°C. To detect and quantify serum cytokines, commercial enzyme-linked immunosorbent assay (ELISA) kits (Thermo Fisher Scientific, Massachusetts, USA) were used, and the assay was performed according to the manufacturers` guidelines and methods. To detect the cytokine levels in serum samples, 1:100 dilution was made in PBS. The MULTISKAN SkyHigh plate reader (Thermo Fisher Scientific) was employed to determine the optical density. The serum cytokines (IFN-γ, TNF-α, IL-2, IL-6, IL-10, and IL-12p70) were quantified by extrapolating the standard curves.

### Preparation of *P. berghei* ANKA lysate

Previously established protocol by (Kim et al., 2022) was used to prepare *P. berghei* ANKA crude antigen. Concisely, *P. berghei* infected-blood was withdrawn intracardially when the parasitemia was more than 30%. Collected blood was centrifuged at 3,000 × g for 5 min and lysed chemically. For lysing, 0.15% saponin in PBS was mixed with equal volume of pelleted RBCs at room temperature for 5 mins. Followed by centrifugation, the pelleted parasites were washed 3 times with 1× PBS and re-suspended in 1× PBS. Released parasites were subjected to mechanical disruption by 2 cycles of sonication for 30 seconds, at 40% amplitude. Protein quantity of the lysate was measured by Pierce™ BCA Protein assay kit (Thermo Fisher Scientific, USA). The lysate was kept at −20°C and later used as a coating agent for ELISA plates.

### Determination of humoral immunity

To assess the humoral immune response, antibodies against *B. microti* and *P. berghei* were measured by ELISA. We had sera from Bm, PbA, Bm/PbA7, and Naïve mice (n=5) collected at day 8 pci. Co-infected mouse serum was detected for antibodies against each parasite. A previously described protocol (Zafar et al., 2022) was followed to measure the antibody response. Either 50 μL *P. berghei* lysate or GST-fused rBmP32 antigen was used to coat microtiter plates (Nunc, Roskilde, Denmark) and kept at 4°C overnight. Washing was performed with 0.05% Tween 20-PBS (PBST) and blocking with 3% skim milk in PBS for 1 h at 37°C. After washing, the plates were incubated with 50 µL of 1:100 diluted serum samples in a blocking solution for 1 h at 37°C. While the samples were put in incubation, goat anti-mouse-Immunoglobulin HRP-conjugated secondary antibody (IgG, IgG1, Ig2a, IgG3, and IgM) were prepared. Secondary antibodies were diluted in a blocking solution to a concentration of 1:4,000. After incubation with diluted mouse serum, plates were washed six times. Then, incubated for 1 h at 37°C with secondary antibody and again washed six times. Finally, plates were incubated with tetramethylbenzidine (TMB) substrate for 1 h at room temperature (RT) to detect bound antibodies. Absorbance values were read at 415 nm using the MULTISKAN SkyHigh plate reader (Thermo Fisher Scientific). To ensure the consistency of results and avoid any discrepancy, triplicates of each sample were used.

### Absolute quantification of *P. berghei* parasites

At day 8 pci, organs were harvested and blood was collected from mouse groups (PbA and Bm/PbA7; n=5). DNA was extracted from tissues and blood using NucleoSpin^®^ Tissue Kit (Takara, Japan), and QIAamp^®^ DNA Blood Mini Kit (Qiagen, Hilden, Germany), respectively, adhering to the directions specified by the manufacturer. The DNA was eluted to a final volume of 100 μL. To quantify the parasites, a pair of specific primers (F: 5`-AAGCATTAAATAAAGCGAATACATCCTTAC-3`; R: 5`GGAGATTGGTTTTGACGTTTATGTG-) was used to amplify a part of the 18S rRNA gene (134 bp) of *P. berghei* (Baptista et al., 2010). A final volume reaction of 10 μL was run in duplicates, composed of 5 μL 1 × PowerUp™ SYBR™ Green Master Mix (Applied Biosystems, Massachusetts, USA), 0.8 μM primers, 1 uL DNA, and 2.4 uL UltraPure™ DNase/RNase-free water (Thermo Fisher Scientific). The cycling regimen employed was 50°C for 2 min, 95°C for 2 min, 40 cycles of 95°C for 15 sec, and 60°C for 45 sec, with a dissociation stage. The reactions were run in the QuantStudio™ 5 Real-time PCR System (Thermo Fisher Scientific). Serially diluted standards prepared from plasmids served as reference samples. Gene copy values were derived from the average quantified cycle values of the replicates relative to the obtained values from the standards. Values were converted to their corresponding log values followed by statistical analysis.

### Evans blue assay for assessment of the BBB disruption and lungs vascular integrity

To measure the disruption of BBB in C57BL/6 mouse groups (n=5), either single infected or challenge-infected with *P. berghei* ANKA and in Naïve, we followed the previously stated protocol by (Baptista et al., 2010). Concisely, 200 μL of 2% Evans blue (EB) in PBS was injected intravenously (iv) in the mouse tail after the onset of clinical symptoms of ECM. One group of Naïve mice was injected with PBS as the negative control to distinguish morphology. One hour later, mice were euthanized and perfused with PBS. Harvested brains and lungs were weighed, photographed, and kept in 2 mL formamide (Merck, New Jersey, USA) for 48 h at 37°C to recover the Evans blue dye from the tissue. The extent of BBB and lung damage was assessed by measuring the absorbance at 620 nm. Evans blue concentration was quantified by calculations using a standard curve.

### Statistical analyses

GraphPad Prism 8 software (GraphPad Prism, California, USA) was used for statistical analyses of all data. To make a comparison among groups, ordinary one-way analysis of variance (ANOVA) followed by *post hoc* Tukey’s multiple-comparisons test and two-tailed unpaired student t-test was used. A P-value less than 0.05 was considered significant at a 95% confidence interval.

## Results

### Extended survival of co-infected mice with *B. microti* primary infection against *P. berghei* deadly challenge

**Figure 1:**
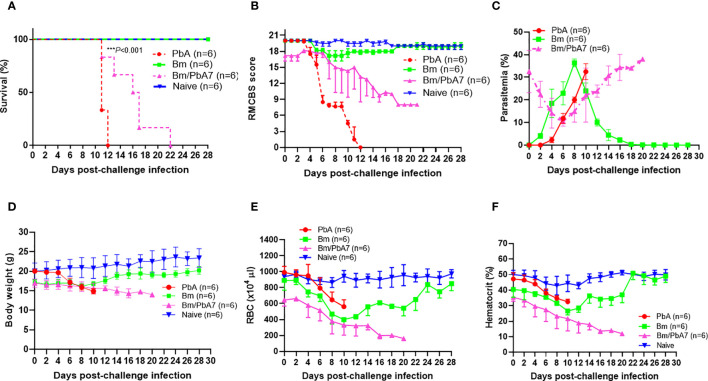
Monitoring the course of disease in C57BL/6J mice in Bm, PbA7, Bm/PbA7, and Naïve mice groups (n=6). In the first trial, test mice were initially infected with *B microti* and then challenge infected with *P. berghei* at day 7 post-primary infection. **(A)** Survival curve, **(B)** RMCBS score, **(C)** course of parasitemia, **(D)** body weight, **(E)** red blood cell (RBC) count, and **(F)** hematocrit values were monitored until PbA and Bm/PbA7 mice died and for 28 days for Bm and Naïve mice. Mean percent parasitemia, body weight, RBC, and hematocrit values were calculated from individual values taken from all surviving mice at each specific time point. Results are expressed as the mean values ± standard deviation (SD) of six mice (n = 6). Ordinary one-way analysis of variance (ANOVA) with Tukey’s test was used for the comparison of parasitemia between Br and co-infected groups, Two-way ANOVA followed by Tukey`s multiple comparison test was used for the analysis of clinical score, whereas for the survival analysis the Kaplan-Meier non-parametric model was used. Asterisks indicate statistical significance (***P < 0.001).

In the current study, Bm/PbA7 mice survived longer than PbA mice. All the PbA mice succumbed to infection within day 12, as evident in **Figure 1A**, whereas Bm/PbA7 mice survived until day 21 and died at day 22 post-challenge infection (pci). Interestingly, PbA mice showed signs of piloerection, shivering, convulsions, seizures, and coma, while co-infected mice survived without any overt signs of ECM (**Figure 1B**). These results demonstrated that, although complete survival was not observed, primary infection with *B. microti* allowed a longer survival than the PbA mice.

### Parasitemia and hematologic indices during acute stage of *B. microti* and *P. berghei* co-infection

Throughout the experiment, levels of parasitemia were monitored by Giemsa-stained blood smears. The curves represented in “**Figure 1C**” were used to compare the parasitemia among the three groups. The comparison revealed that at day 0 of post-challenge infection, the Bm/PbA7 mice had a parasitemia level of 32.5% resulting from a previous *B. microti* infection. Subsequently, the parasitemia declined by day 6 post-challenge infection (pci) (which means day 13 post-primary infection), but it later peaked again. This subsequent increase in parasitemia was attributed to the *P. berghei* challenge-infection. It is important to note that the observed parasitemia represents combined parasitemia caused by both *Babesia* and *Plasmodium* parasites. Surprisingly, co-infected mice showed the highest parasitemia (38%) while the peak parasitemia in PbA mice was 32.47%. In the case of Bm mice, the peak parasitemia level reached 36.39% and was observed on day 8 post-infection (pi), (**Figure 1C**). The body weights of the Bm mice showed transient loss that was recovered after the parasitemia was resolved. In PbA mice, drastic weight loss was seen from initial weight (20.058 g) to final weight (14.887 g) before they expired, whereas gradual decline in weight was seen in co-infected mice from (16.98 g) to (14 g) (**Figure 1D**). Moreover, all groups developed anemia, especially the Bm and Bm/PbA7 mice, as evidenced by lower-than-normal RBCs and hematocrit indices. Before the day of death, Bm/PbA7 mice exhibited intense anemia, whereas hematologic parameters were rehabilitated and sustained until day 28 pci in Bm mice (**Figures 1E, F**) (Zafar et al., 2022). Our results confirmed that escalation in blood parasites expedited the breakdown of iRBCs and reduced hematologic indices, resulting in anemia.

### Immune microenvironment in the spleen, brain, liver, lungs, and kidneys of mice

**Figure 2:**
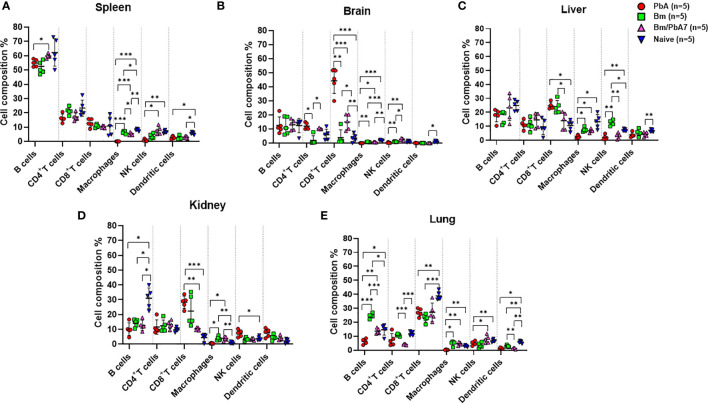
A subsequent trial was conducted to perform immunophenotyping of different types of immune cells in mice spleen, brain, liver, kidney, and lungs at day 8 post-challenge infection based upon surface markers by FACS. **(A)** Spleen **(B)** Brain, **(C)** Liver, **(D)** Kidney, and **(E)** Lungs in PbA, Bm, Bm/PbA7, and Naïve mice (n=5). Markers used were CD45+ CD3+ cells (T cells), CD45+ CD19+ cells (B cells), CD45+ CD49b+ cells (natural killer cells), CD45+ CD11c+cells (dendritic cells), and CD45+ F4/80+ cells (macrophages). Analysis of variance (ANOVA) was followed by *post hoc* Tukey’s multiple comparison test. The percentage population of each cell type is presented as mean ± SD. Asterisks indicate statistical significance (* *P* < 0.05; **P < 0.01; ***P < 0.001).

We probed the above-mentioned crucial organs by flow cytometry to shed light on the immune cell dynamics. The aim was to study the impact of co-infection on immune cell population during early stage infection, and hence determine the role of innate immunity in our study. Spleen, brain, liver, lungs, and kidneys were aseptically removed and, after processing the samples, data was collected (**Figure 2**). Although the brain of PbA mice showed the lowest population of NK cells, DCs, and macrophages, the population of CD4+ and CD8+ T cells significantly increased compared to the other groups (**Figure 2B**). Co-infected mice had significantly higher number of B cells than PbA mice (**Figure 2A**). There were marked differences in the macrophage population between PbA and Bm/PbA7 mice. Macrophages were more predominant immune cells of all the probed organs in co-infected mice compared to PbA mice. Notably, macrophages were significantly higher in co-infected (5.36%) spleen compared to PbA (0.13%) mouse spleen (**Figure 2A**). Among all organs, the liver had the greatest percentage of macrophages in all the groups: 2.56%, 7.41%, 6.67%, and 13.27% in PbA, Bm, Bm/PbA7, and Naïve mice, respectively (**Figure 2C**). PbA mice had a comparatively lower immune cell population in kidneys and lungs than Bm/PbA7 mice (**Figures 2D, E**). Percentages of B cells, CD4+ and CD8+ T cells, macrophages, NK cells, and DCs varied among all groups, with spleen of PbA mice being the lowest in immune cell population (except for CD8+ cells) and Bm/PbA7 being comparatively higher (**Supplementary Table 2**).

### The interplay between pro-inflammatory and anti-inflammatory cytokines might drive the outcome of *B. microti* and *P. berghei* co-infection in mice

**Figure 3:**
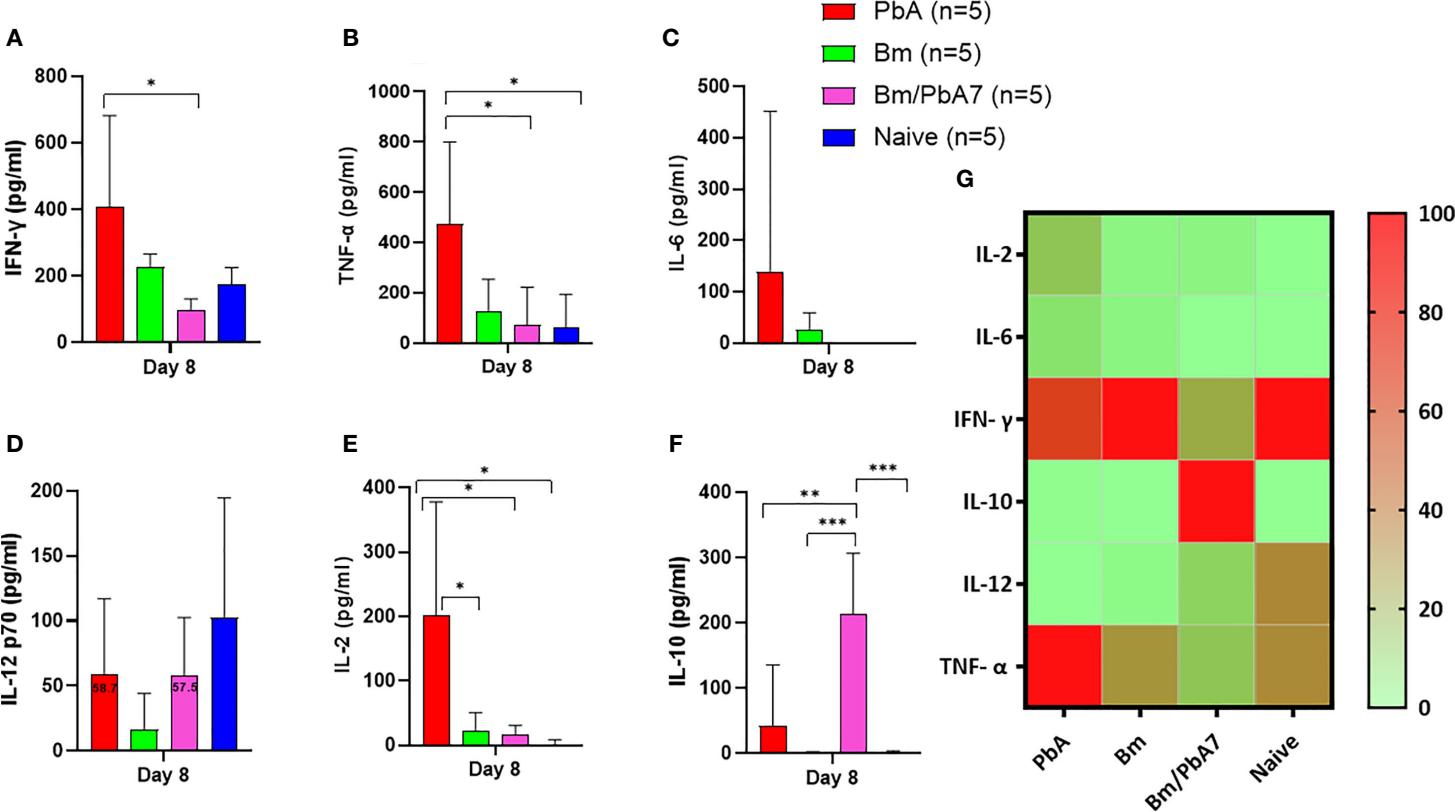
The kinetics of serum cytokines of experimental PbA, Bm, Bm/PbA7, and Naïve mice groups (n=5). Test C57BL/6J mice were initially infected with *B microti* and then challenge infected with *P. berghei* at day 8 post-primary infection. Serum was collected from all groups at day 8 pci and levels of **(A)** IFN- g, **(B)** TNF-a, **(C)** IL-6, **(D)** IL-12p70, **(E)** IL-2, and **(F)** IL-10 were measured. **(G)** Heatmap representing the progression of secretion of the six cytokines. The results are expressed as means ± SD. Ordinary one-way analysis of variance (ANOVA) with Tukey’s test was used for the statistical analysis. Asterisks denote statistical significance (*P < 0.05; **P < 0.01; ***P < 0.001).

In an attempt to comprehend the cytokine crosstalk in mice, we measured the serum cytokine levels (IFN-γ, TNF-α, IL-6, IL-12p70, IL-2, and IL-10) (**Figure 3G**). Co-infected mice exhibited decreased levels of pro-inflammatory cytokines (i.e., IFN-γ, TNF-α, and IL-12p70), whereas the level of anti-inflammatory cytokine (IL-10) was elevated in the acute phase of co-infection (**Figure 3**). Although the decrease in the level of IL-12p70 was not statistically significant, it is worth noting that PbA mice exhibited a slightly higher level compared to co-infected mice: 58.7 pg/mL in PbA mice and 57.5 pg/mL in Bm/PbA7 mice. Our findings suggested that IL-10 plays a protective role against ECM as the coinfected mice had significantly higher levels of IL-10 compared to PbA mice (**Figure 3F**). Furthermore, the absence of early secretion of IFN-γ resulted in deadly infection as significantly higher levels of IFN-γ were seen in PbA mice at day 8 pi (**Figure 3A**) compared with Bm/PbA7 mice. Relatively higher levels of TNF-α and IL-12p70 were seen in the PbA mice, however, a significant difference was not seen in the levels of IL-12p70 (**Figures 3B, D**). In PbA mice, the level of TNF-α in serum was enhanced in comparison to other groups (**Figure 3B**). The raised levels of IL-6 (**Figure 3C**) and IL-2 (**Figure 3E**) were also noticed in mice with *P. berghei* infection. Altogether, our findings suggest that IL-10, IFN-γ, and TNF-α are key cytokines as high levels of IFN-γ and TNF-α contributed to the lethality of disease and IL-10 acted as a protector in the course of *B. microti* and *P. berghei* co-infection.

### Modification of humoral response during *B. microti* and *P. berghei* co-infection

**Figure 4:**
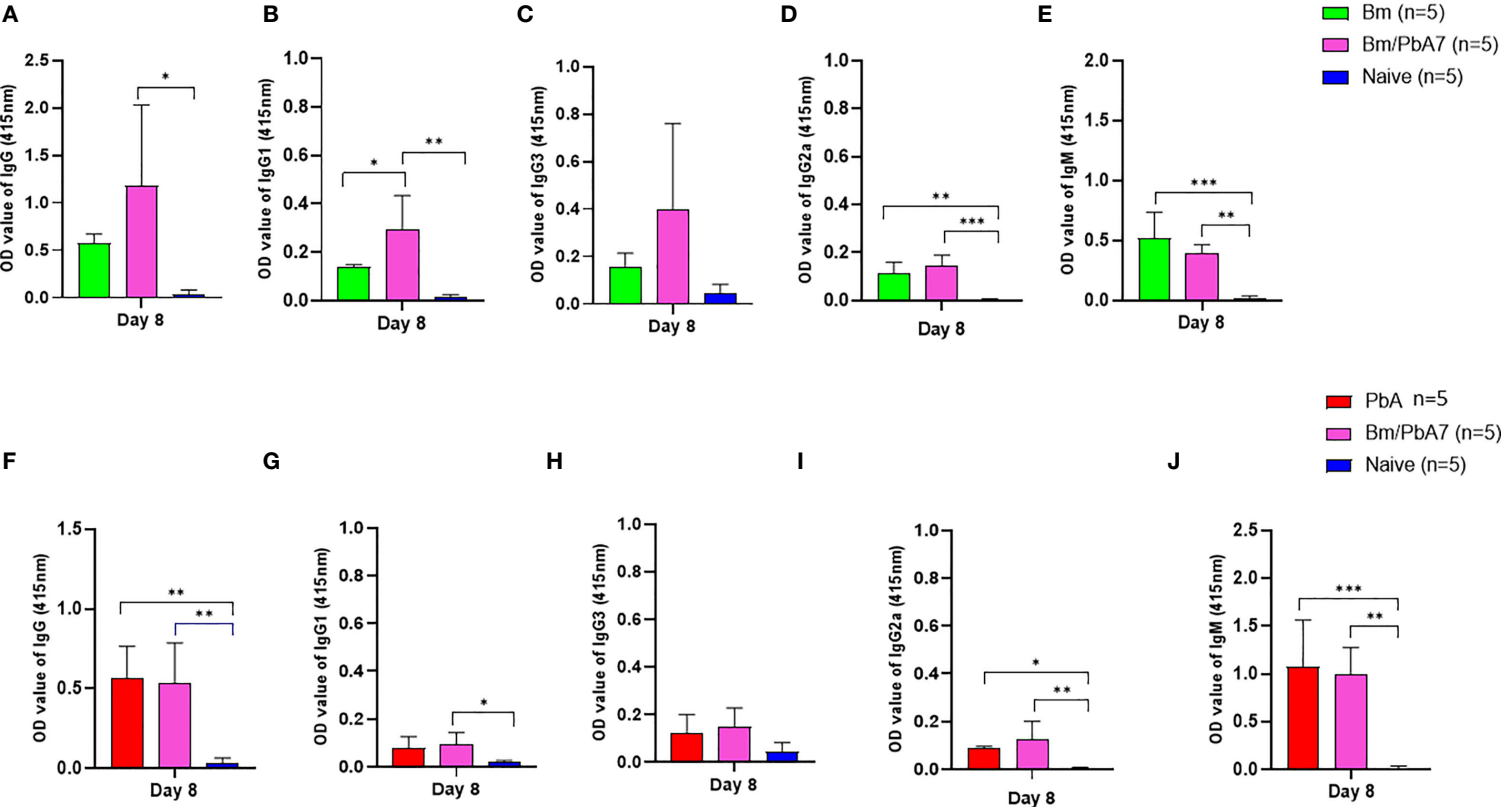
The dynamics of serum antibodies against *B microti* and *P. berghei* in Bm, PbA mice, Bm/PbA7, and Naïve mice groups (n=5). The production of **(A)**, IgG **(B)**, IgG1 **(C)**, IgG3 **(D)**, IgG2a and **(E)**, IgM showed *B microti-*specific while **(F)**, IgG **(G)**, IgG1 **(H)**, IgG3 **(I)**, IgG2a and **(J)**, IgM showed *P. berghei-*specific antibodies in mouse serum collected at day 8 pci post-challenge infection. Results of ELISA indicated a significant increase in the specific antibodies in the serum of co-infected mice. Ordinary one-way analysis of variance (ANOVA) with Tukey’s test was used for the statistical analysis. Asterisks indicate statistically significant differences (*, *P* < 0.05; **, *P* < 0.005; ***, *P* < 0.0001. The results are expressed as mean values ± the SD for five mice.

Later, we aimed to validate if the spike in B and T cells corroborated the (**Figure 2**) species-specific antibody response. Therefore, antibodies against *B. microti* and *P. berghei* (**Figure 4**) were evaluated at day 8 pci. For single-infected mice, day 8 pci was day 8 post-infection while for co-infected mice it was day 14 post-*B. microti* primary infection and day 8 pci at the time of serum collection. Therefore, the co-infected mice had pronounced IgG-specific antibody responses against *B. microti* in mice (**Figure 4A**). Although the levels of IgM in single-infected Bm and PbA mice were higher compared to co-infected mice, the difference was not found to be statistically significant (**Figures 4E, J**). The specific antibody production against *P. berghei* was comparable between co-infected and PbA mice. Our results suggested that there was not much difference between serum antibody production in PbA and co-infected mice. Overall, our results showed that mice during the acute stage infection had lowered humoral immune response.

### Alterations in structure of organs during acute stage of *Babesia* co-infection

**Figure 5:**
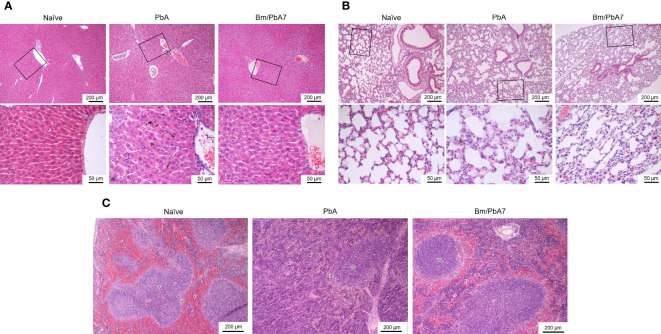
Histopathological analysis of the **(A)** liver, **(B)** lung, and **(C)** spleen depicting the impact of *P. berghei* and Bm/PbA7 mice compared to Naïve mice (n=1). Sections of 5 μm thickness were collected and stained with H & E Black boxes indicate **(A)** Naïve mice showed liver lobules with an orderly arrangement of hepatocyte plates. This arrangement and morphology of hepatocytes were severely damaged in PbA mice livers, while in Bm/PbA7 mice morphology was normal. **(B)** Lung of Naïve mice had intact structure, containing pulmonary alveoli separated by thin septa. PbA mice showed severe and moderate interstitial pneumonia, with the thickening of the interstitium. Mild abnormality was seen in the Bm/PbA7 mice. **(C)** Naïve mice spleen showed distinct red (r) and white (w) pulps, PbA mice indicated the blurred boundary between red and white pulp because of repletion of immune cells in the red pulp. In Bm/PbA7 mice spleen, the marginal zone (*) is distinguished by the white pulp clearly.

In H & E-stained sections of the spleen, Naïve mice had typical, normal histology with distinct red and white pulps (**Figure 5C**). In the spleens infected with *P. berghei* ANKA, the boundary between red and white pulps became blurred because of repletion of immune cells in the red pulp, whereas, in the co-infected spleen, the development of the marginal zone distinguished the white pulp more clearly (**Figure 5C**). In the liver of Naïve mice, normal structure, which consists of liver lobules with an orderly arrangement of hepatocyte plates, was observed (**Figure 5A**). This arrangement and morphology of hepatocytes were severely damaged in the livers infected with *P. berghei* ANKA, whereas in the liver of co-infected mice these were normal (**Figure 5A**). The lung of Naïve mice had intact and typical structure and normal histology, which contains pulmonary alveoli separated by thin septa (**Figure 5B**). The lungs infected with *P. berghei* ANKA showed severe and moderate interstitial pneumonia, with the thickening of the interstitium. This abnormality became mild in the co-infected lung (**Figure 5B**). Overall, these findings implied that co-infection lessened the disease manifestations in the spleen, liver, and the lungs of mice.

### *B. microti* primary infection reduces subsequent tissue sequestration of *P. berghei*

**Figure 6:**
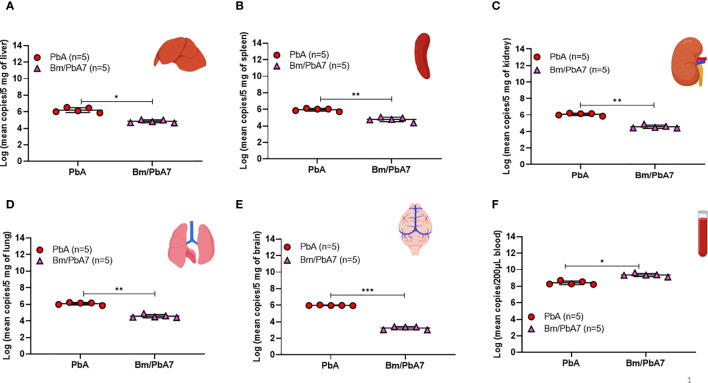
Measure of *P. berghei* burden in mice **(A)** Liver **(B)** Spleen, **(C)** Kidney, **(D)** Lung, **(E)** Brain, and **(F)** Blood of PbA and Bm/PbA7 mice (n=5). Mean copy numbers of *P. berghei* 18S in mouse DNA samples (n = 5 per group at day 8 post-challenge infection) were transformed to log values. Bm/PbA7 mice had less sequestration of *P. berghei* in organs than PbA mice. Individual values are the mean of duplicate samples. Log values were analyzed using one-way ANOVA and Tukey’s multiple comparison *post hoc* test; ***P < 0.001.

The results of flow cytometry suggested the possible role of innate immune cells in protective immunity (**Figure 2**), likewise, our qPCR results pointed out the fact that innate immune cells played a defensive role against *P. berghei* infiltration in organs as evidenced by the low *P. berghei* burden in tissues including liver, spleen, kidneys, lungs, and brain. Thus, to corroborate whether severe disease manifestation in PbA mice is exacerbated due to the accumulation of *P. berghei* iRBCs in organs, qPCR was performed on DNA extracted from the brain, liver, lungs, spleen, kidneys, and blood of PbA and co-infected mice at day 8 pci (**Figure 6**). We found the extent of tissue injury (**Figure 5**) was directly proportional with *P. berghei* sequestration in the tissues, with significantly lower *P. berghei* burden in tissues including liver, spleen, kidneys, lungs, and brain (**Figures 6A-E**). Surprisingly, mouse blood showed the opposite result, with higher parasite load in co-infected mice compared to PbA mice (**Figure 6F**). *P. berghei* load was increased in organs of PbA mice as compared to co-infected mice. These results clearly established that the protective immune response led to the evasion of sequestration of high load of iRBCs in co-infected mice. Low parasite burden in liver, spleen, kidney, lung, and brain of co-infected mice is concomitant with the prevention of tissue injury (**Figures 6A–E**). These results were statistically significant.

### Disruption of BBB and lung vascular permeability is concomitant with ECM in PbA mice

**Figure 7:**
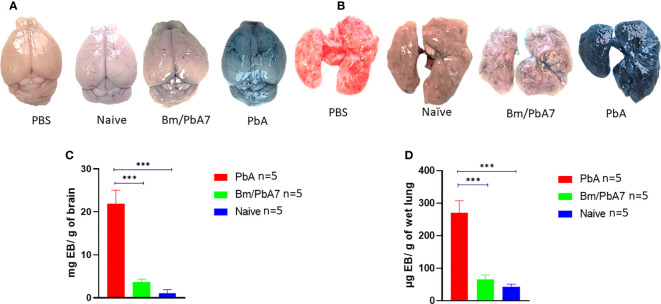
Second trial was conducted employing PbA, Bm/PbA7, and Naïve mice (n=5). Following the protocol as described in the method section, harvested brains and lungs from mice were weighed and photographed. The morphology and color of the **(A)** brain and **(B)** lungs. The amount of Evans blue dye that infiltrated the **(C)** brain and **(D)** lungs was measured. Co-infected mice showed less extravasation of Evans blue dye in the brain and lungs as compared to the PbA mice. Analysis of variance (ANOVA) followed by *post hoc* Tukey’s multiple comparison test. Results are expressed as means ± SD. Asterisks indicate statistical significance (***P < 0.001).

To ascertain that co-infected mice died later without ECM, evaluation of the integrity of the BBB and changes in lung vascular endothelial barrier function was assessed by measuring Evans blue infiltration in respective tissues. As anticipated, the integrity of BBB and lung vascular endothelial barrier was present in co-infected mice, whereas in PbA mice, the BBB became more permeable with onset of ECM (**Figures 7A, B**). Comparatively higher extravasation of Evans blue was noted in PbA mice in contrast to co-infected mice (**Figure 7C**). Overall, our results suggest that the severity of CM characterized by the compromised BBB caused by *P. berghei* infection dramatically decreased in co-infected mice. In addition, function of the lung vascular endothelial barrier was more impaired in PbA mice in comparison to co-infected mice as indicated by higher infiltration of Evans blue dye (**Figure 7D**).

## Discussion

We were interested in investigating co-infections and assessing whether they played a part in the development or evasion of complicated malaria and related pathologies. Evidence of brain, liver, lung, and spleen pathology, as well as anemia and tissue-sequestration of parasites, have been reported in mice (Briquet et al., 2015). Our research demonstrated the modification of disease outcomes and immune responses in a mouse co-infection model. In Bm/PbA7 mice, high levels of parasitemia at day 0 of challenge-infection due to *B. microti* were considered advantageous for early activation of the innate immunity. Furthermore, *P. berghei* has a preference for reticulocytes. It is believed that this preference is responsible for the increased parasite burden leading to severe anemia, and vice versa (Thakre et al., 2018). Eventually, we observed Bm/PbA7 mice succumbed to infection due to anemia. The sequestration of *Plasmodium* in tissues is supposed to be beneficial to the parasite, because it avoids the recognition and clearance of iRBCs by macrophages in the spleen (Lagassé et al., 2016). The sequestration of iRBCs is evidently linked to pulmonary pathology in the murine model (Franke-Fayard et al., 2005; Lovegrove et al., 2008). The infection of hepatocytes by the sporozoites of *Plasmodium* can result in congestion, sinusoidal blockage, and cellular inflammation (Al-Salahy et al., 2016). Our results also showed that the intensity of malaria-induced lung pathologic features was proportional to the increased levels of iRBCs sequestration. This finding was in agreement with Lagassé et al. (2016). Organ damage observed in PbA mice, including brain, liver, and lungs, was a consequence of diminished microcirculatory blood flow caused by structural and functional changes of iRBCs (Dondorp et al., 2000). Thus, inadequacy of antimalarial immunity by *Plasmodium* may be clarified due to the evasion of the immunological armamentarium by infiltration in organs and reduced immunogenicity (Dondorp et al., 2000). A significant characteristic of severe malarial syndrome is that parasite loads are significantly elevated compared to individuals afflicted with uncomplicated malaria. Hence, this indicated that parasite load played a pivotal role in developing brain, lung, and liver disease during malaria (Bagot et al., 2002; Haque et al., 2011; Strangward et al., 2017). Even though the peripheral parasitemia was higher in Bm/PbA7 mice, our qPCR results indicated less sequestration of *P. berghei* in the brain, liver, lungs, spleen, and kidney as compared to PbA mice. This might explain the prolonged survival and evasion of severe malaria in Bm/PbA7 mice as compared to PbA mice.

Macrophages were identified as the most crucial cell types associated with the host immune response against malaria infections. Besides the phagocytosis of iRBCs, they also affect adjacent tissue environment by releasing pro-inflammatory and anti-inflammatory cytokines (Ozarslan et al., 2019). Mice devoid of macrophages exhibited more malaria complications, like disruption in the BBB and increased vascular permeability of the lungs (Gupta et al., 2016). These studies insinuated an imperative role of macrophages in the immunity against malaria and that their activation can modulate outcome of the disease. However, the mechanisms modulating their activation are not known. With a few exceptions, innate immune cell (B cells, NK cells, DCs, and macrophages) populations in PbA mice organs plummeted, whereas those of co-infected mice organs spiked (**Supplementary Table 2**). Although *Plasmodium* causes immunosuppression (Millington et al., 2007), *B. microti* primary infection mice seemed to evade the immunosuppressive effect of *Plasmodium* in co-infected mice. Thus, depending on the organ, the innate immune response decides the fate of infection. This explains the prolonged survival and partial protection of co-infected mice as we exposed the mice to a *Plasmodium*-like parasite to activate the first line of defense. There is an agreement that the presence of CD8+ T cells and iRBC sequestration in the brain is crucial for the progress of ECM. Activation of brain endothelial cells could phagocytize the pathogen and present antigen to CD8+ T cells. This would also start to kill the endothelial cells via the perforin pathway by CD8+ T cells, which could result in BBB breakdown (Baptista et al., 2010). Additional studies advocated that infiltration of CD8+ T and CD4+ T cells in the brain is a hallmark of cerebral pathology (Grau et al., 1986; Belnoue et al., 2002). Liver damage takes place in ECM before the onset of neurological symptoms. Hence, the liver is considered as a prominent depot of CD8+ T cells during the symptomatic stages of ECM (Haque et al., 2011). Here, consistent with our flow cytometric and qPCR analyses, PbA mice had significantly higher infiltration of CD8+ T cells in the brain, liver, and lungs while co-infected mice showed low infiltration of CD8+ T cells and iRBCs. Thus, not only the parasite burden but also CD8+ T cells are important to aggravate multiorgan injury in PbA mice.

Changes in the balance of pro-inflammatory and anti-inflammatory responses can determine the outcome of the disease (Beiting, 2014). IL-10 secretion is a key feature of tissue-resident macrophages (Gupta et al., 2016; Kumar et al., 2019). Complications of malaria are increased in the absence of macrophages (Ozarslan et al., 2019). Stimulation of B cells or infection with *B. microti* resulted in the production of IL-10. The numbers of IL-10-producing B cells also elevated with *B. microti* infection (Jeong et al., 2012). In malaria, tissue is protected from injury by IL-10 production. It inhibits inflammation by suppressing pro-inflammatory cytokine secretion, and also by decreasing the expression of MHC-II molecules on antigen presenting cells (APCs) (Sanni et al., 2004; Couper et al., 2008; Kumar et al., 2019). A low pro-inflammatory response may cause unrestrained replication of parasites, whereas an excess pro-inflammatory response may result in tissue injury. Overall, our cytokine results showed that IL-10 can protect against ECM (Freitas Do Rosario and Langhorne, 2012). Our FACS data confirm these findings as indicated by the significant decline in B cell and macrophage population in PbA mice which led to the decrease in production of IL-10, whereas B cell and macrophages increased in co-infected mice with a concomitant rise in the level of IL-10 cytokine.

Early secretion of IFN-γ can safeguard from ECM (King and Lamb, 2015), whereas the absence of timely release of IFN-γ can lead to lethality (Stevenson and Riley, 2004). IL-10 hampered the excess secretion of IFN- γ and TNF-α during *B. microti* infection (Djokic et al., 2018), while strengthening the immune response to eliminate the parasite (Trinchieri, 2007; Couper et al., 2008; Redpath et al., 2014). Pathology in malaria is related to the excess production of IFN-γ and IL-12 (Engwerda et al., 2002). In another study, protected mice depicted insignificant amounts of IFN-γ and TNF-α and increased concentration of IL-10 post-*P. chabaudi* challenge infection. The cytokine results likely indicated that the safeguard provided by *B. microti* against *P. berghei* is due to a lack of high levels of IFN-γ, TNF-α, and IL-12p70. On the other hand, levels of these cytokines peaked in PbA mice, in contrast to co-infected mice. A possible mechanism by which pro-inflammatory mediators such as TNF-α and IFN-γ cause tissue pathology is by increasing the adhesion molecules, such as ICAM-1, VCAM-1, and P-selectin on the endothelial cells leading to the accumulation of leukocytes and iRBC, inferring that iRBC and leukocyte sequestration may play a pathogenic role (Bagot et al., 2002; Claser et al., 2019).

Our study has certain limitations. One limitation is that we did not utilize a green fluorescent protein (GFP) parasite, which would have enabled us to distinguish the impact of co-infection on PbA parasitemia alone. Additionally, employing a GFP parasite would have facilitated convenient visualization and tracking of the parasite’s location and behavior within tissues. Another area that warrants investigation is the effect of co-infection on the prepatent period. In contrast to the previous study (Efstratiou et al., 2020), which showed 100% cross-protection by *B. microti* against lethal *P. chaubadi*, our study suggests primary infection with *B. microti* is linked to mitigating the disease manifestations in complicated malaria compared to sole infection with *P. berghei*. Since the co-infected mice did not show complete cross-protection, we need to explore other factors that might enhance the immune response and further strengthen the host defense. To further establish the protective role of macrophages in controlling and resisting infections, it is necessary to transfer the bone marrow-derived macrophages (BMM) from naive mice to co-infected mice. This approach will enable us to determine whether the co-infected mice exhibit enhanced survival, along with improved recovery from parasitemia, compared to PbA mice that did not receive BMM. Additional investigation will also be needed to decipher the role of distinct macrophage subtypes, such as M1 and M2, in orchestrating the equilibrium between pro- and anti-inflammatory cytokines, which we propose to facilitate cross-protection.

## Conclusions

Herein, we used a murine co-infection model of severe malaria. Our results demonstrated that the primary *B. microti* infection prolonged the survival in co-infected mice and lessened the severity of disease compared to *P. berghei* control mice. Furthermore, we quantified and compared immune cell compositions of the liver, lungs, kidneys, brain, and spleen of all experimental groups of mice, which provided a detailed insight into the immune microenvironment inside organs. These data indicate that innate immunity is critical for cross-protection and macrophages appear to play a role in conferring immunity in co-infected mice. In addition to higher parasite burden in the organs, a high population of CD8^+^T cells was associated with the organ damage. Co-infected mice exhibited decreased levels of pro-inflammatory cytokines (i.e. IFN-γ, TNF-α, and IL-12p70), in contrast to the level of anti-inflammatory cytokine IL-10, which was boosted in mouse serum, suggesting a protective function of IL-10 against complicated malaria. Further research is required to investigate the malaria-suppressing effects of *Babesia* to get a better understanding, with an aim to develop novel therapeutic tools to control malaria.

## Data availability statement

The original contributions presented in the study are included in the article/**Supplementary Material**. Further inquiries can be directed to the corresponding authors.

## Ethics statement

The animal study was reviewed and approved by Research Ethics Review Committee of the Obihiro University of Agriculture and Veterinary Medicine.

## Author contributions

IZ, and XX planned this project. IZ executed all experiments. DK performed the histopathology. TT, EMG, SJ, SAE-SE-S, TD, HL, MMA, MZ, and MY facilitated in performing the experiments. IZ, HBB, MAR, EMG, JZ, and NI evaluated the results and wrote the manuscript. XX revised it and all authors endorsed the revised manuscript.
