# Supplementary material for: Babesia microti alleviates disease manifestations caused by Plasmodium berghei ANKA in murine co-infection model of complicated malaria
Source: Front Cell Infect Microbiol. 2023 Jul 10;13:1226088. doi: 10.3389/fcimb.2023.1226088 (PMC10364126; doi:10.3389/fcimb.2023.1226088)
Supplement: Supplementary file 1 [file DataSheet_1.pdf]

frontiers

Supplementary Material

Babesia microti Alleviates Disease Manifestation Caused by *Plasmodium berghei* ANKA in Murine Co-infection Model of Complicated Malaria

Iqra Zafar[1,2], Tomoyo Taniguchi[3], Hanadi B. Baghdadi[4,5], Daisuke Kondoh[6], Mohamed Abdo Rizk[7], Eloiza May Galon[1,8], Shengwei Ji[1], Shimaa Abd El-Salam El-Sayed[1,9], Thom Do[1], Hang Li[1], Moaz M. Amer[1], Maa Zhowei[1], Ma Yihong[1], Jinlin Zhou[10], Noboru Inoue[1], Xuenan Xuan[1*]

*** Correspondence:**
Xuenan Xuan
gen@obihiro.ac.jp

Supplementary Table 1.1. Antibody panel 1 used for FACS analyses

Immune cells	Anti-mouse antibody markers	Fluorophore	Antibody dilution (vol/vol µL)	Manufacturer (catalog no.)
Total leukocytes	CD45	FITC	0.5/200	BioLegend (147709)
T lymphocytes	CD3	PE/Cyanine7	1/200	BioLegend (100219)
B lymphocytes	CD19	Brilliant Violet 421™	1/200	BioLegend (115537)
Natural killer cells	CD49b	PerCP/Cyanine5.5	1/200	BioLegend (108915)
CD4 cells	CD4	PE	1/200	BioLegend (100408)
CD8 cells	CD8a	Brilliant Violet 510™	1/200	BioLegend (100751)

Supplementary Table 1.2. Antibody panel 2 used for FACS analyses

Immune cells	Anti-mouse antibody markers	Fluorophore	Antibody dilution (vol/vol µL)	Manufacturer (catalog no.)
Total leukocytes	CD45	FITC	0.5/200	BioLegend (147709)
Macrophages	F4/80	PE	1/200	BioLegend (123109)
Dendritic cells	CD11c	Brilliant Violet 510 ™	1/200	BioLegend (117337)

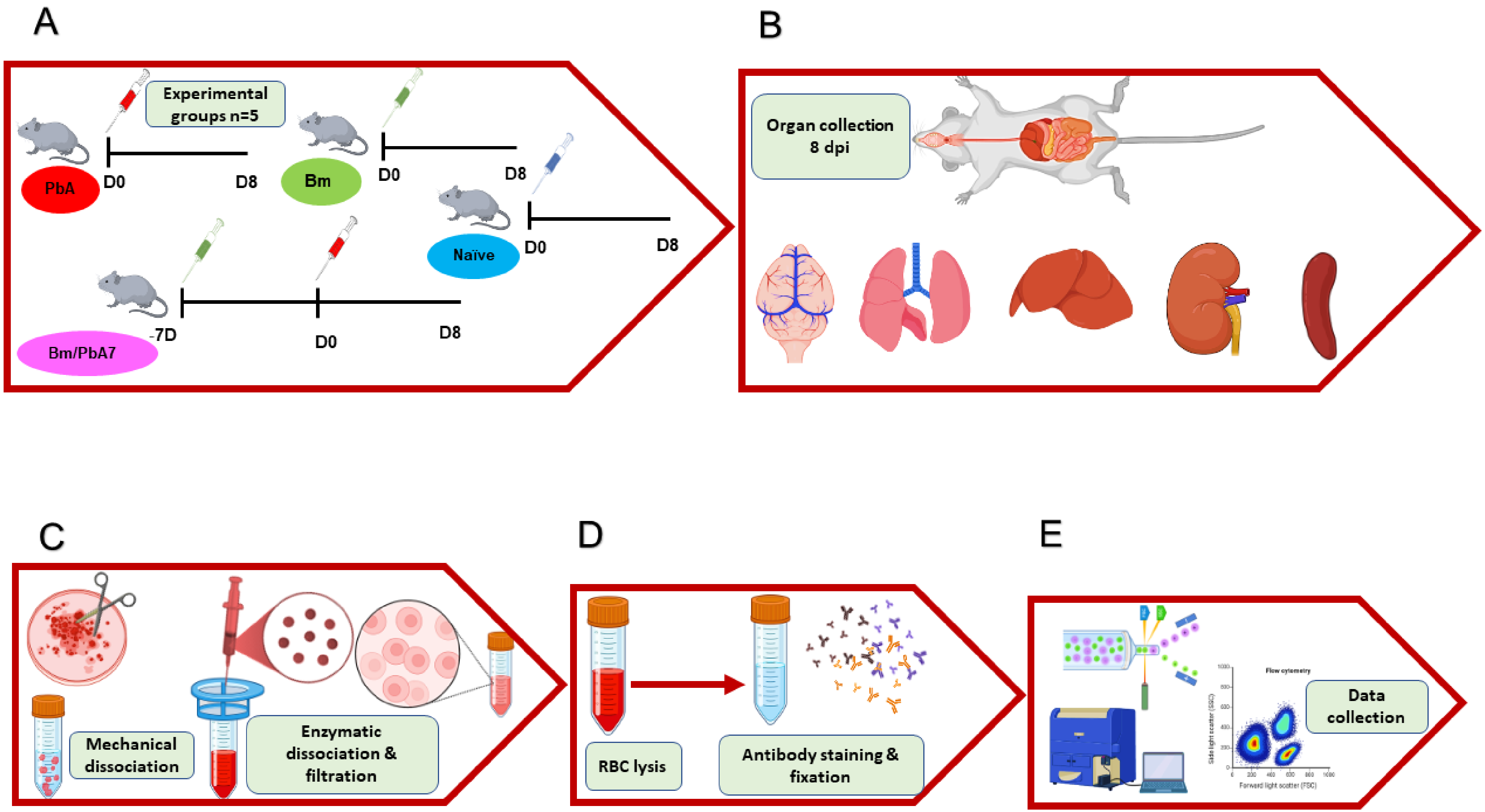

Supplementary Figure 1. Figure showing processes involved in performing FACS. (A) Overall experiment design, (B) Organ harvest at day 8 post challenge-infection (pci) from all groups (PbA, Bm, Bm/PbA7 and naïve, (C) Mechanical and enzymatic dissociation and filtration, (D) RBC lysis, antibody staining and fixation, (E) Data collection by flow cytometer and analysis of data.

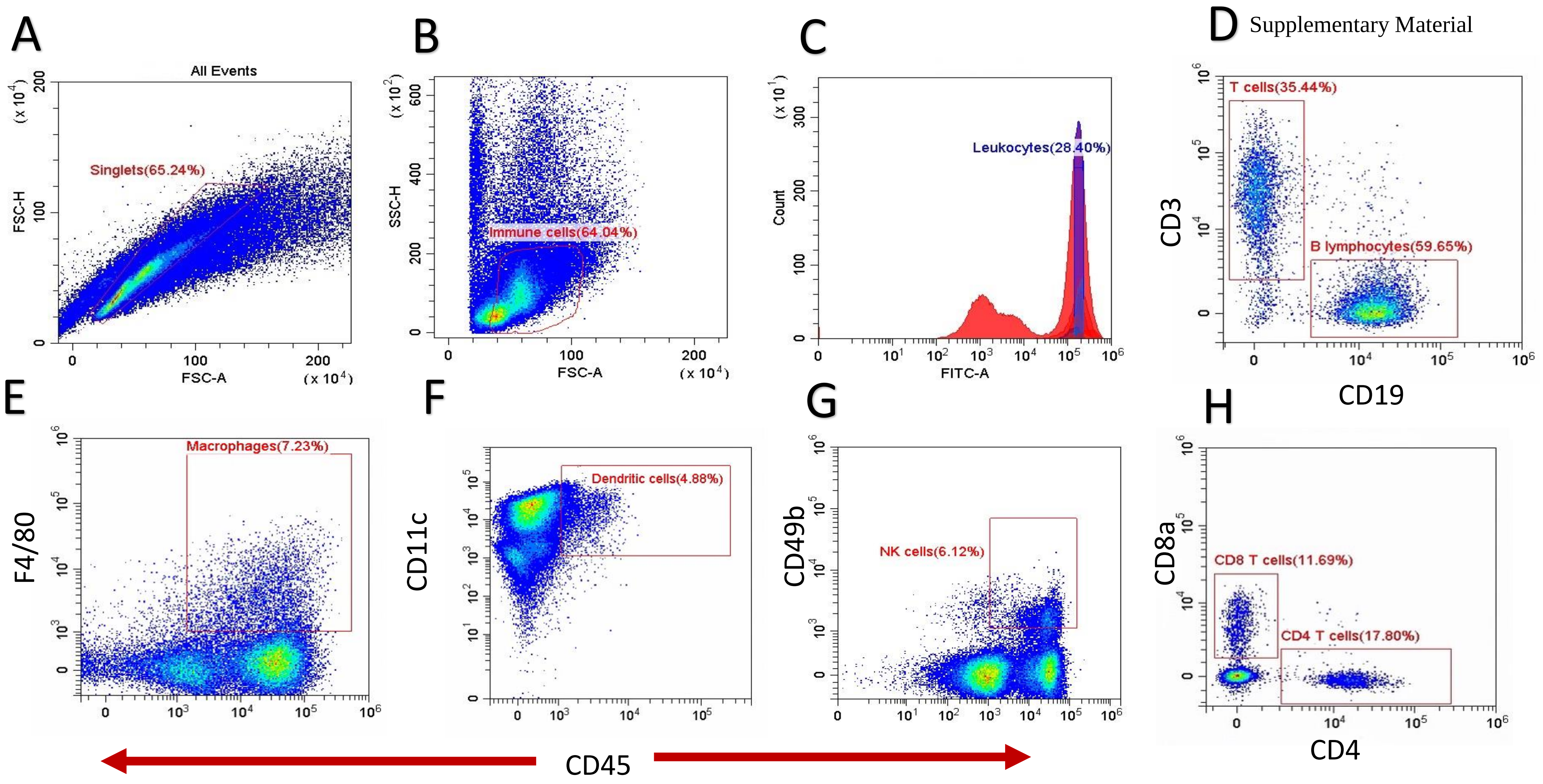

Supplementary Figure 2. Gating scheme for fluorescence-activated sorting (FACS) analysis of immune cells. A total of 50,000 events were analyzed. Each panel is a representative image of gating for (A) singlets, (B) $CD45^{+}$ (immune cells), (C) $CD45^{+}$ leukocytes, (D) $CD45^{+}$ $CD3^{+}$ (T cells), $CD19^{+}$ (B cells), (E) $CD45^{+}$ $F4/80^{+}$ (macrophages), (F) $CD45^{+}$ $CD11c^{+}$ (dendritic cells), (G) $CD45^{+}$ $CD49b^{+}$ (natural killer cells), and (G) $CD45^{+}$ $CD8a^{+}$ (CD8 T cells), $CD45^{+}$ $CD4^{+}$ (CD4 T cells).

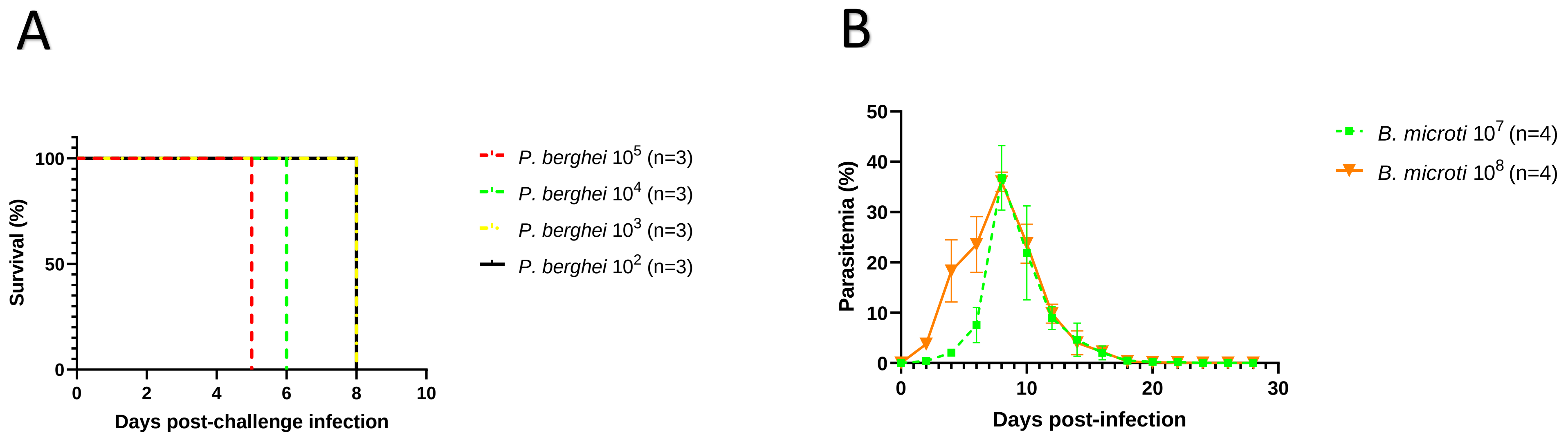

Supplementary Figure 3. Results of preliminary trials. (A) Out of the four doses (10^2,103, 10^4, 10^5), 10^3, was selected for *P. berghei* infection. (B) For *B. microti* infection, two doses were used in the preliminary trial, and out of those, 10^8 was selected for primary infection.

Supplementary Table 2. Percentage of immune cell populations in test organs.

ORGANS	IMMUNE CELLS%	PbA	BM	BmPbA7	Naïve
Spleen	B lymphocytes	54.688	53.463	59.760	62.256
	CD8	13.332	10.207	10.280	10.886
	CD4	16.967	21.680	17.144	23.338
	NK cells	1.042	3.203	7.490	6.734
	Dendritic cells	2.794	2.113	2.932	5.762
	Macrophages	0.138	6.433	5.366	8.134
Brain	B lymphocytes	12.642	12.304	13.100	12.496
	CD8	44.378	3.982	15.048	3.602
	CD4	12.034	1.962	10.220	5.966
	NK cells	0.348	0.988	0.504	1.079
	Dendritic cells	0.028	0.030	0.048	1.116
	Macrophages	0.078	0.780	0.705	2.020
Liver	B lymphocytes	17.622	16.464	23.154	24.776
	CD8	24.602	23.838	14.050	10.506
	CD4	11.068	11.200	14.474	9.640
	NK cells	1.954	12.360	2.956	7.176
	Dendritic cells	3.748	5.200	4.076	6.750
	Macrophages	2.560	7.416	6.676	13.270
Lung	B lymphocytes	6.290	24.538	13.610	14.598
	CD8	26.794	22.856	27.012	38.860
	CD4	7.398	10.700	4.148	11.918
	NK cells	5.054	3.142	5.168	1.796
	Dendritic cells	1.432	11.501	1.204	1.846
	Macrophages	0.322	4.882	4.480	2.884
Kidney	B lymphocytes	10.072	14.152	12.598	30.958
	CD8	11.496	22.274	28.270	4.456
	CD4	8.578	12.200	13.108	9.782
	NK cells	7.272	3.374	3.360	3.788
	Dendritic cells	7.544	4.500	3.924	2.070
	Macrophages	0.506	3.646	3.725	0.840
